# Supplementary material for: Local Health Department COVID-19 Vaccination Efforts and Associated Outcomes: Evidence from Jefferson County, Kentucky
Source: Vaccines (Basel). 2025 Aug 26;13(9):901. doi: 10.3390/vaccines13090901 (PMC12474317; doi:10.3390/vaccines13090901)
Supplement: Supplementary file 1 [file vaccines-13-00901-s001.zip › vaccines-3742020-supplementary.pdf]

Table S1. COVID-19 Outcomes and LMPHW-Coordinated Doses per 100,000 Residents in Jefferson County.

	Overall	
	N	[2]Rate per 100,000
[1]Reported COVID-19 Positive Cases	148,429.00	18,157.65
[1]COVID-19 Related Hospitalizations	4,605.00	563.34
[1,3]COVID-19 Related Deaths	1,402.00	171.51
LMPHW Event Doses	14,324.00	1,752.29

[1]Outcome totals reflect cumulative counts from December 2020 to May 2022.
[2]Rates per 100,000 were calculated using the 2021 Jefferson County population estimate (N = 817,446, Source: U.S. Census Bureau).
[3]LMPHW event doses include only those administered through events coordinated by the Louisville Metro Public Health and Wellness Department.

Table S2. Associations between the Number of COVID-19 Vaccine Doses Administered at the LMPHW-Coordinated Events and COVID-19 Outcomes in Jefferson County, Kentucky (Unadjusted Regression Models).

	One-Week Lag	Two-Week Lag	Three-Week Lag	Four-Week Lag	One-Month Lag
[1]COVID-19-related Deaths					
[2]Number of Doses	-0.001	-0.000	0.000	-0.000	-0.000
	(0.001)	(0.001)	(0.001)	(0.001)	(0.001)
[1]COVID-19-related Hospitalizations					
[2]Number of Doses	0.005***	0.008***	0.006***	0.008***	0.013***
	(0.002)	(0.002)	(0.002)	(0.002)	(0.004)
[1]COVID-19 Positive Cases					
[2]Number of Doses	-0.026	-0.015	0.005	0.029	0.027
	(0.067)	(0.067)	(0.068)	(0.068)	(0.188)

[1]Dependent variables

[2]COVID-19 vaccine doses coordinated by the Louisville Metro Public Health and Wellness Department (LMPWH)

Standard errors in parentheses

*** $p<0.01$

Table S3. Associations between the Number of COVID-19 Vaccine Doses Administered at the LMPHW-Coordinated Events and COVID-19 Outcomes in Jefferson County, Kentucky (Unadjusted Fixed-Effects Regression Model: Race).

	One-Week Lag	Two-Week Lag	Three-Week Lag	Four-Week Lag	One-Month Lag
**White**					
[1]COVID-19-related Deaths					
[2]Number of Doses	-0.001	-0.001	-0.000	-0.000	-0.001
	(0.001)	(0.001)	(0.001)	(0.001)	(0.001)
[1]COVID-19-related Hospitalizations					
[2]Number of Doses	0.003**	0.005***	0.003**	0.006***	0.008**
	(0.001)	(0.001)	(0.001)	(0.001)	(0.003)
[1]COVID-19 Positive Cases					
[2]Number of Doses	-0.043	-0.037	-0.021	-0.006	-0.031
	(0.052)	(0.052)	(0.053)	(0.053)	(0.148)
**Black**					
[1]COVID-19-related Deaths					
[2]Number of Doses	-0.000	-0.000	-0.000	-0.000	-0.000
	(0.000)	(0.000)	(0.000)	(0.000)	(0.000)
[1]COVID-19-related Hospitalizations					
[2]Number of Doses	0.002***	0.003***	0.003***	0.002***	0.005***
	(0.001)	(0.001)	(0.001)	(0.001)	(0.001)
[1]COVID-19 Positive Cases					
[2]Number of Doses	0.017	0.022	0.025	0.031**	0.054
	(0.015)	(0.015)	(0.015)	(0.015)	(0.038)
**Other**					
[1]COVID-19-related Deaths					
[2]Number of Doses	-0.000	0.000	0.000	0.000	0.000
	(0.000)	(0.000)	(0.000)	(0.000)	(0.000)
[1]COVID-19-related Hospitalizations					
[2]Number of Doses	0.000**	0.000	0.001***	-0.000	0.001
	(0.000)	(0.000)	(0.000)	(0.000)	(0.000)
[1]COVID-19 Positive Cases					
[2]Number of Doses	0.001	-0.000	0.001	0.003	0.004
	(0.005)	(0.005)	(0.005)	(0.005)	(0.013)

[1]Dependent variables
[2] COVID-19 vaccine doses coordinated by the Louisville Metro Public Health and Wellness Department (LMPWH)
Standard errors in parentheses
*** p<0.01, ** p<0.05,

Table S4. Associations between the Number of COVID-19 Vaccine Doses Administered at the LMPHW-Coordinated Events and COVID-19 Outcomes in Jefferson County, Kentucky (Poisson Regression).

	One-Week Lag	Two-Week Lag	Three-Week Lag	Four-Week Lag	One-Month Lag
[1]COVID-19-related Deaths					
[2]Number of Doses	-0.003***	-0.002***	-0.001	-0.001	-0.003***
	(0.001)	(0.001)	(0.001)	(0.001)	(0.001)
[1]COVID-19-related Hospitalizations					
[2]Number of Doses	-0.001	0.001	-0.001	0.001	-0.001
	(0.001)	(0.001)	(0.001)	(0.001)	(0.001)
[1]COVID-19 Positive Cases					
[2]Number of Doses	-0.091***	-0.078***	-0.052***	-0.025***	-0.155***
	(0.005)	(0.005)	(0.005)	(0.005)	(0.009)

[1]Dependent variables

[2]COVID-19 vaccine doses coordinated by the Louisville Metro Public Health and Wellness Department (LMPWH)

Standard errors in parentheses

*** $p<0.01$

Table S5. Associations between the Number of COVID-19 Vaccine Doses Administered at the LMPHW-Coordinated Events and COVID-19 Outcomes in Jefferson County, Kentucky (Negative Binomial Regression).

	One-Week Lag	Two-Week Lag	Three-Week Lag	Four-Week Lag	One-Month Lag
[1]COVID-19-related Deaths					
[2]Number of Doses	-0.003***	-0.001**	-0.001	-0.001	-0.006
	(0.001)	(0.001)	(0.001)	(0.001)	(0.002)
[1]COVID-19-related Hospitalizations					
[2]Number of Doses	-0.000	0.002	0.000	0.002	0.006
	(0.001)	(0.001)	(0.001)	(0.001)	(0.004)
[1]COVID-19 Positive Cases					
[2]Number of Doses	-0.147***	-0.135***	-0.130***	-0.088	-0.326***
	(0.045)	(0.046)	(0.049)	(0.050)	(0.159)

[1]Dependent variables

[2]COVID-19 vaccine doses coordinated by the Louisville Metro Public Health and Wellness Department (LMPWH)

Standard errors in parentheses

*** p<0.01, ** p<0.05

Table S6. Associations between the Number of COVID-19 Vaccine Doses Administered at the LMPHW-Coordinated Events and COVID-19 Outcomes in Jefferson County, Kentucky (Multiple Linear Regression with all Time-Lags).

	One-Week Lag	Two-Week Lag	Three-Week Lag	Four-Week Lag
[1]COVID-19-related Deaths				
[2]Number of Doses	-0.002***	-0.001**	-0.000	-0.000
	(0.001)	(0.001)	(0.001)	(0.001)
[1]COVID-19-related Hospitalizations				
[2]Number of Doses	-0.003	0.000	-0.001	0.001
	(0.002)	(0.002)	(0.002)	(0.002)
[1]COVID-19 Positive Cases				
[2]Number of Doses	-0.077	-0.082	-0.039	-0.007
	(0.078)	(0.077)	(0.077)	(0.077)

[1]Dependent variables

[2]COVID-19 vaccine doses coordinated by the Louisville Metro Public Health and Wellness Department (LMPWH)

Standard errors in parentheses

*** $p<0.01$, ** $p<0.05$

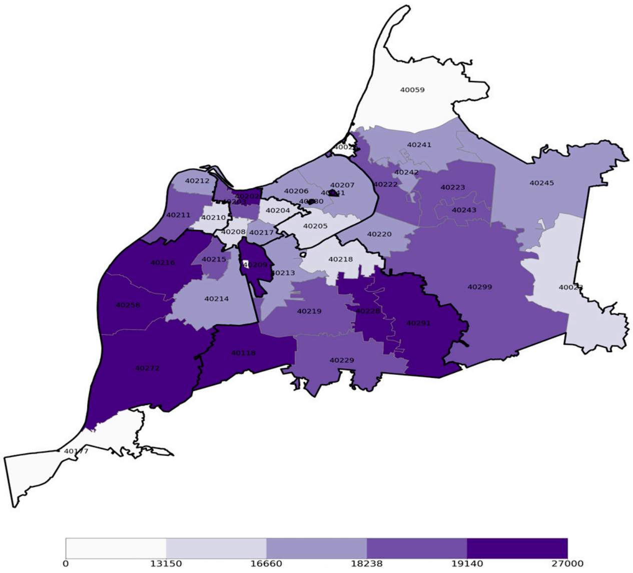

Figure S1. COVID-19 Reported Cases per 100,000 Residents by ZIP Code, Jefferson County, December 2020–May 2022.

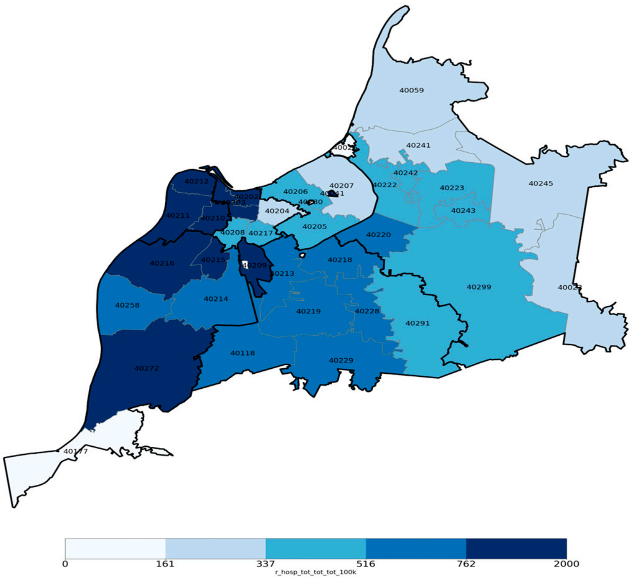

Figure S2. COVID-19-Related Hospitalizations per 100,000 Residents by ZIP Code, Jefferson County, December 2020–May 2022.

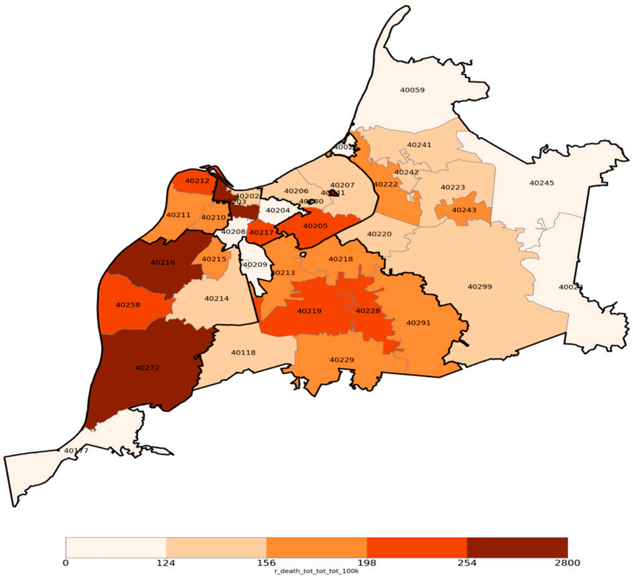

Figure S3. COVID-19-Related Deaths per 100,000 Residents by ZIP Code, Jefferson County, December 2020–May 2022.

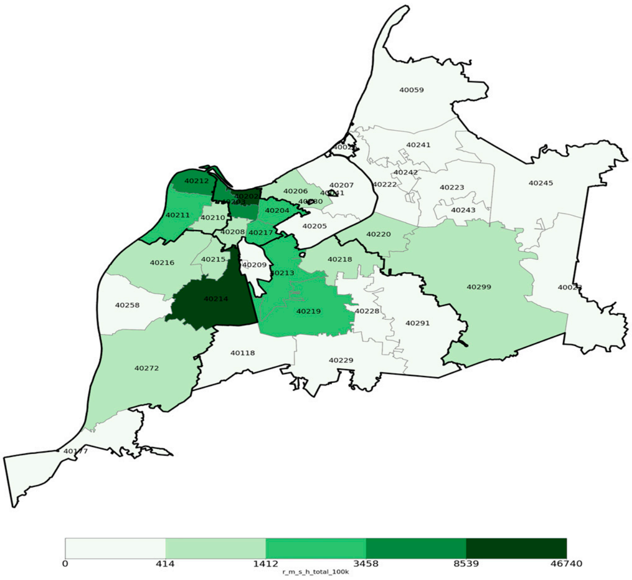

Figure S4. LMPHW-Coordinated COVID-19 Vaccine Doses per 100,000 Residents by ZIP Code, Jefferson County, December 2020–May 2022.
